# Maternal plasma vitamin D levels across pregnancy are not associated with neonatal birthweight: findings from an Australian cohort study of low-risk pregnant women

**DOI:** 10.1186/s12884-022-05336-0

**Published:** 2023-01-26

**Authors:** Paige F. van der Pligt, Stacey J. Ellery, Deborah L. de Guingand, Gavin Abbott, Paul A. Della Gatta, Robin M. Daly

**Affiliations:** 1 Institute for Physical Activity and Nutrition (IPAN), School of Exercise and Nutrition Sciences, Deakin University, Geelong, Australia; 2 Department of Nutrition, Western Health, Footscray, VIC Australia; 3 The Ritchie Centre, Hudson Institute of Medical Research, Clayton, VIC Australia; 4 Department of Obstetrics and Gynaecology, Monash University, Clayton, VIC Australia

**Keywords:** Vitamin D, Pregnancy, Birthweight, Macrosomia, Birth size, Obesity

## Abstract

**Background:**

In utero environments can be highly influential in contributing to the development of offspring obesity. Specifically, vitamin D deficiency during pregnancy is associated with adverse maternal and child health outcomes, however its relationship with offspring obesity remains unclear. We assessed maternal vitamin D status across pregnancy, change in plasma vitamin D concentrations and associations with neonatal birthweight, macrosomia and large for gestational age.

**Methods:**

Women (*n* = 221) aged 18–40 years with singleton (low-risk) pregnancies, attending antenatal clinics at a tertiary-level maternity hospital were recruited at 10–20 weeks gestation. Medical history, maternal weight and blood samples at three antenatal clinic visits were assessed; early (15 ± 3 weeks), mid (27 ± 2 weeks) and late (36 ± 1 weeks) gestation. Maternal 25(OH)D was analysed from stored plasma samples via liquid chromatography-tandem mass spectrometry (LC/MS/MS). Neonatal growth parameters were collected at birth. Unadjusted and adjusted linear and logistic regression assessed associations of maternal vitamin D with birthweight, macrosomia and large for gestational age.

**Results:**

Mean plasma 25(OH)D increased from early (83.8 ± 22.6 nmol/L) to mid (96.5 ± 28.9 nmol/L) and late (100.8 ± 30.8 nmol/L) gestation. Overall 98% of women were taking vitamin D-containing supplements throughout their pregnancy. Prevalence of vitamin D deficiency (25(OH)D < 50 nmol/L) was 6.5%, 6.3% and 6.8% at early, mid and late pregnancy respectively. No statistically significant association was found between 25(OH)D or vitamin D deficiency at any timepoint with neonatal birthweight, macrosomia or large for gestational age.

**Conclusions:**

Prevalence of vitamin D deficiency was low in this cohort of pregnant women and likely related to the high proportion of women taking vitamin D supplements during pregnancy. Maternal 25(OH)D did not impact offspring birth weight or birth size. Future studies in high-risk pregnant populations are needed to further assess maternal vitamin D status and factors in utero which promote early life obesity.

**Supplementary Information:**

The online version contains supplementary material available at 10.1186/s12884-022-05336-0.

## Background

Prevalence of childhood overweight and obesity are increasing globally [1, 2] and macrosomia (birthweight > 4000 g) [3, 4] and large for gestational age (LGA) [5] are established factors which independently predict risk of future adiposity and early childhood obesity [6–8]. Over the past decade, there has been an upwards trend of increasing birthweight across developed countries [9], with a number of modifiable factors such as maternal obesity, poor diet quality and physical inactivity during pregnancy having been shown to impact propensity for high birthweight, macrosomia and offspring adiposity [10–12]. Assessment of factors in utero which might predispose offspring to high birthweight is a necessary step in understanding maternal–fetal interactions and may inform novel and much needed approaches to understanding predictors of offspring overweight and obesity [1].

Emerging evidence has shown that vitamin D deficiency (VDD) (25-hydroxyvitamin D concentration (25(OH)D) below 50 nmol/L (20 ng/mL)) [13] during pregnancy increases the risk for multiple adverse obstetric and neonatal health outcomes [14–16]. Immune regulation of pregnancy progression is largely dependent on vitamin D homeostasis during pregnancy [17] and adequate maternal vitamin D during pregnancy is critical to meet fetal calcium demands [18] and support healthy embryonic development [19]. Pregnant women are considered a ‘high risk’ group for VDD with an estimated prevalence of 50—80% [20–22] and low levels of maternal vitamin D during pregnancy have been associated with impaired fetal growth [23], including low birthweight (LBW) and small for gestational age (SGA) births [19, 24–27].

Given that evidence points to vitamin D playing a role in increasing lipolysis and fatty acid oxidation while decreasing adipogenesis [28], and that VDD in adults has been associated with obesity [29, 30] and metabolic syndrome [28, 30], exploring the role of maternal vitamin D in development of neonatal birthweight and birth size is an important step in assessing and further understanding modifiable risk factors for early life obesity. Recently, some evidence has emerged showing that low maternal vitamin D levels are associated with increased offspring adiposity in childhood and adolescence [31], although the causal relationship is unclear [31]. Some studies have also shown levels of maternal 25(OH)D to be significantly lower in LGA babies, compared to babies born SGA [32] and cord blood 25(OH)D to be significantly lower in LGA neonates compared to those born between 3000 g – 4000 g [33]. Yet, conflicting results have been observed overall [24, 34] and variation in diagnostic measurement of 25(OH)D, lack of longitudinal assessment of maternal 25(OH)D across pregnancy [19], variation in gestational week of sampling and adjustment for seasonal variation may in part have contributed to inconsistent results [34].

Whilst only a very small proportion of vitamin D is obtained via dietary sources including oily fish and eggs, vitamin D produced endogenously from sunlight is the most important and abundant source, stimulating production of 1,25-dihydroxyvitmain D_3_ (the biologically active form of vitamin D) through a two-step process of hydroxylation within the liver and then the kidneys. Women born in Asia, the Middle East and Africa are widely recognised as being at ‘high risk’ for VDD [15, 35] due to a range of biological and cultural characteristics. Factors including dark skin pigmentation [36] and wearing of covered clothing due to religious reasons [37] can contribute to women from these regions being more vulnerable to VDD during pregnancy. Plasma concentrations of vitamin D can further vary depending on latitude and air pollution [17], but vitamin D supplementation during pregnancy is recognised as a safe and effective approach to prevent deficiency [38]. The objectives of this study were to assess maternal vitamin D status across pregnancy, evaluate changes in plasma 25(OH)D concentrations with pregnancy progression and assess associations with neonatal birthweight, macrosomia and LGA, in a sample of low-risk pregnant women in Australia.

## Methods

### Study design and sampling

This was a retrospective longitudinal study involving women with low-risk pregnancies, and involved secondary analysis of data collected as part of the Creatine in Pregnancy Outcomes study (CPO) [39]. Further details of the CPO study protocol, recruitment of women and methodology have been reported previously [39]. Briefly, we analysed data from a sample of 221 women included in the original CPO study who attended low-risk antenatal clinics and were planning to give birth at Monash Health in Melbourne, Australia. Monash Health provides antenatal healthcare to more than 9,000 women each year across South-East Melbourne and is one of the largest maternity providers in the state of Victoria [40]. Women were recruited from antenatal clinics between 10–20 weeks gestation and at consent elected to have samples, collected as part of the CPO study, stored as a biobank for future research approved by Monash Health. Ethical approval for the original CPO study was granted in August 2015 from Monash Health (Ref 14140B) and Monash University (Ref 7785) and the current study was granted ethical approval in July 2109 by the Deakin University Human Research Ethics Committee (DUHREC) (2019–282). All methods were carried out in accordance with organisational ethical guidelines and regulations and informed consent was received prior to participation in the study as per ethical regulations.

### Inclusion / exclusion criteria

Women were included in the CPO study if they were aged 18–40, with a singleton pregnancy and were between 10–20 weeks gestation at recruitment. Women needed to be classified as having a ‘low-risk’ pregnancy, meaning they had no known pre-existing medical or obstetric condition. Women were excluded if they were non-English speaking, had a non-singleton pregnancy, had been previously diagnosed with Type 1 or Type 2 diabetes, were taking creatine supplements during pregnancy, disclosed ongoing alcohol or illicit drug use during pregnancy, or who were not attending their antenatal appointments as part of routine hospital care. The original CPO study included data for 284 women collected from 2015 to 2017. A minimum two blood samples collection at two different visits for plasma 25(OH)D analyses was set as criterion for inclusion into the current study.

### Measurement of study variables

This study used health and medical history data and blood samples collected at three time-points (mean ± SD); 15 ± 3 weeks gestation, 27 ± 2 weeks gestation and 36 ± 1 weeks gestation. We defined these timepoints as *early* pregnancy, *mid* pregnancy and *late* pregnancy, respectively. Demographic data included maternal age, education, parity, smoking status and sex of the baby. Region of birth was defined using the categories: Australian / New Zealand (Australia and New Zealand); Asian / South Asian (India, Nepal, China, Afghanistan, Singapore, Sri Lanka, Philippines, Malaysia, Pakistan, Bangladesh, Thailand); European / UK / Canada (Russia, Switzerland, Ireland, UK, Canada, Greece, France, Scotland, Poland, Germany, England); Middle Eastern / African/ South American (Lebanon, Israel, Kuwait, Iran, Venezuela, Mauritius, Ghana, Ethiopia, Colombia). The categories Asian / South Asian and Middle Eastern / African / South American were combined to define a ‘high risk’ cultural group for VDD, as women born in these regions are considered at higher risk for deficiency [15, 36]. Medical history data (e.g. pre-existing medical conditions and gestational diabetes diagnosis (GDM)) was recorded form hospital medical records and detail regarding whether women were taking a vitamin D-containing supplements (no supplement; vitamin D only; a pregnancy multivitamin (contains some vitamin D but a lower dose than ‘vitamin D only’ supplements); vitamin D supplement plus a pregnancy multivitamin) were collected at the first antenatal visit.

### Anthropometry

Women’s height (cm) at the first antenatal clinic visit and weight (kg) using calibrated industrial scales at each clinic visit were measured by trained researchers. Maternal gestational weight gain (GWG) was calculated as weight at late pregnancy minus weight at early pregnancy and body mass index (BMI) (kg/m^2^) was calculated by using World Health Organisation (WHO) BMI criteria, to categorise women as either healthy weight, overweight or obese [41]. Neonatal growth parameters were recorded at delivery and all outcomes reported in this study were extracted by trained, hospital-based researchers from the Monash Health Birthing Outcomes System (BOS) database, which uses a standardized method of reporting perinatal data in Victoria, Australia. Birthweight > 4000 g was used to classify macrosomia as is standard criteria and widely accepted [42–44]. Low birth weight was defined at < 2500 g as is also standard criteria [45]. For babies born full term, WHO growth percentiles were used to define SGA (< 10^th^ percentile at birth) [45, 46] and LGA (> 90^th^ percentile at birth) [42]. Fenton Growth Charts for Preterm Infants (< 37 weeks) [47, 48] were used to classify preterm babies (*n* = 9) as SGA and LGA.

### Plasma 25-hydroxyvitamin D

A databank and biobank of blood samples were collected at the five antenatal clinic visits as part of the original CPO study. Since few studies have assessed longitudinal maternal 25(OH)D concentrations [49], we deemed assessment of plasma vitamin D at three antenatal visits suitable for this study, which enabled change in concentration across early, mid and late pregnancy to be assessed. For each woman, blood samples at a first (early pregnancy), third (mid pregnancy) and fifth (late pregnancy) antenatal visit were prioritised. If samples were not available at the third or fifth visit, samples were analysed from visit four or five. It was important to allow maximum time between samples analysed given the half-life of plasma 25(OH)D can be up to 25 days [50, 51] and up to 82 days following supplementation with vitamin D [51, 52].

Plasma 25(OH)D was assessed by liquid chromatography-tandem mass spectrometry (LC/MS/MS) using a Shimadzu Nexeria ultra performance LC system with a Sciex 3200QTRAP (SCIEX, Concord, ON, Canada) at Monash Health Pathology. The intra-assay CV was 5.8% at 15.6 nmol/L, 6.0% at 58.0 nmol/L and 3.0% at 140 nmol/L and the inter-assay CV was %. 7.2% at 34.9 nmol/L, 6.9% at 150 nmol/L. The LC/MS/MS method is considered the global reference standard for quantifying 25(OH)D [53, 54]. Vitamin D deficiency was defined as plasma 25(OH)D < 50 nmol/L as per cut-points across multiple countries [55–57] and defined by the Endocrine Society Task Force on vitamin D [13].

### Statistical analyses

Data were analysed using STATA/SE statistical software version 16.0. Descriptive analyses were used to summarise maternal demographics, neonatal outcomes and maternal vitamin D levels, and are reported as either mean ± SD or n (%). For the 221 women in the analysis sample, multiple imputation by chained equations was used to impute missing data (50 imputed datasets), and inferential analyses were conducted by combining estimates from each of the imputed datasets using Rubin’s rules. Under the data missing at random assumption, multiple imputation analyses are known to provide less biased estimates than available or complete case analysis [58]. Available case analyses using the un-imputed data were also conducted as a sensitivity analysis. Associations between vitamin D exposure variables (maternal plasma 25(OH)D (nmol/L) and VDD) at early, mid and late pregnancy and continuous (birth length and weight, maternal GWG and BMI) and binary (macrosomia, LGA) outcomes were tested by fitting both unadjusted and adjusted linear and logistic regression models, respectively using well established known confounders and covariates used previously in assessment of maternal vitamin D and neonatal outcomes [19, 22, 34, 59]. We adjusted for region of birth in all models. For neonatal length and birthweight outcomes, maternal age, gestational length and smoking were additionally adjusted for; for macrosomia and LGA outcomes, maternal age and early pregnancy BMI were additionally adjusted for [43]. For maternal GWG and final BMI outcomes, early pregnancy BMI and education were additionally adjusted for [60–62]. Season of 25(OH)D sample collection was defined as either summer (December-February), autumn (March–May), winter (June–August) or spring (September–November). The average ultra violet index (UBI) measures for summer, autumn, winter and spring in Melbourne across the two-year time period of data collection were 10.0, 4.6, 2.2 and 6.5 respectively [63] (Supplementary data). However, season was not found to be associated with vitamin D exposure variables (data not shown) so was therefore not adjusted for in the final analyses. Statistical significance was set as *p* < 0.05.

## Results

### Participants

Of 273 women who were eligible to be included in the analyses, fifty-one women were not included due to missing data for vitamin D at all three timepoints (*n* = 34) and/or, all child outcomes (*n* = 31) and/or, all demographic data (*n* = 42). This left available data for 222 women. One woman was further excluded as she was taking glucocorticoid steroid medication but not vitamin D supplements, which would likely impact 25(OH)D levels [64]. Data were included for 221 women, of which 7 women had one available vitamin D sample measured (due to insufficient sample available for two further measures), 92 women had two samples measured and 122 women had all three samples measured.

Demographic characteristics of the study cohort are presented in Table 1. The mean age of the women was 31.5 years. Just over half (56.1%) were born in Australia or New Zealand. On average, women delivered at 39.2 weeks gestation. Almost half of the women (46.4%) were first time mothers and three quarters (77.1%) were tertiary educated. Almost all women (96.4%) were non-smokers. Mean maternal BMI was 25.2 kg/m^2^ at early pregnancy and 29.2 kg/m^2^ at late pregnancy. Mean GWG was 11.0 kg and 9.6% of women were diagnosed with GDM during their pregnancy. An approximately equal proportion of babies born were male (51.6%) and female (48.4%) and mean birthweight was 3401.7 g. In total 12.7% of babies were born macrosomic, 12.2% were LGA, 3.6% of babies had low birth weight and 7.2% were born SGA.

**Table 1:** Demographic characteristics of the study cohort

Characteristics	Mean ± SD or n (%)
Maternal age (years)	31.5 ± 3.9
Gestation at delivery (*n* = 220)	39.2 ± 1.6
Parity (*n* = 211)	
1	98 (46.4%)
2	88 (41.7%)
3	25 (11.8%)
Education (*n* = 179)	
Tertiary	138 (77.1%)
Sub-tertiary	41 (22.9%)
Region of birth	
Australia / New Zealand	124 (56.1%)
Asia/ South Asia	65 (29.4%)
Europe / UK / Canada	19 (8.6%)
Middle East / Africa / South America	13 (5.9%)
Smoking	
Non-smoker	213 (96.4%)
Current smoker	8 (3.6%)
Early pregnancy BMI (kg/m^2^)	25.2 ± 4.7
Late pregnancy BMI (kg/m^2^) (*n* = 220)	29.2 ± 4.8
GWG (kg) (n = 220)	11.0 ± 4.6
Diagnosed with GDM (*n* = 208)	20 (9.6%)
Sex of baby	
Male	114 (51.6%)
Female	107 (48.4%)
Birth weight (g)	3401.7 ± 540.7
Birth length (cm) (*n* = 216)	50.0 ± 2.5
Macrosomia	28 (12.7%)
LGA	27 (12.2%)
Low birth weight	8 (3.6%)
SGA	16 (7.2%)

*n* = 221 unless otherwise specified; BMI (body mass index); GWG (gestational weight gain); GDM (gestational diabetes mellitus); LGA (large for gestational age); SGA (small for gestational age)

### Maternal vitamin D and use supplements

**Fig. 1:**
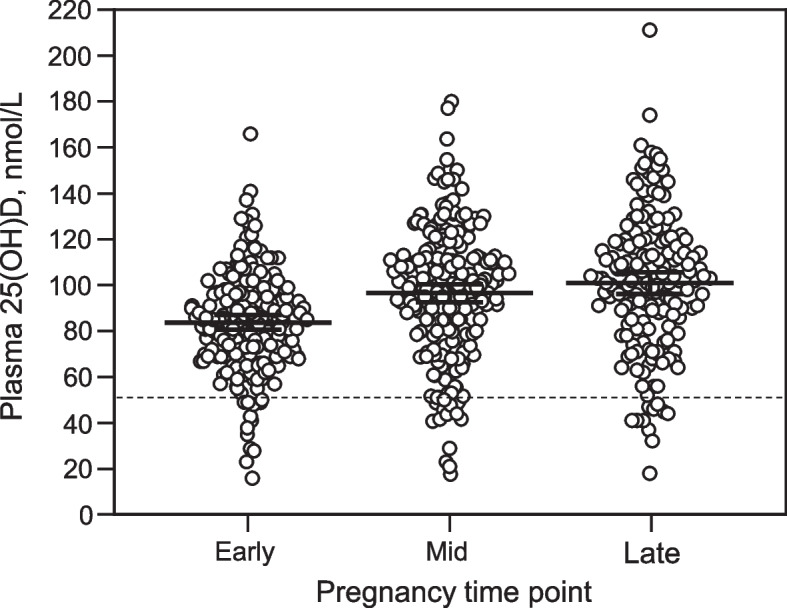
Individual and mean (95% CI) levelsª of maternal 25(OH)D at early, mid and late pregnancy

Data summarising individual maternal vitamin D levels and mean (95% CI) levels across pregnancy are presented in Fig. 1. Mean (95% CI) 25(OH)D levels at early, mid and late pregnancy were 83.8 nmol/L (80.5–87.0), 96.5 nmol/L (92.5–100.4) and 100.8 nmol/L (96.0–105.6) respectively. Differences in 25(OH)D levels at early, mid and late pregnancy and use of vitamin D supplements is presented in Table 2. There was a statistically significant increase in mean 25(OH)D levels from early pregnancy (83.8 nmol/L) to mid pregnancy (96.5 nmol/L) and late pregnancy (100.8 nmol/L). Overall, 6.5%, 6.3% and 6.8% of women were vitamin D deficient at early, mid and late pregnancy, respectively. Of the total number of women who were deficient at early, mid and late pregnancy (*n* = 36), eight women were deficient at multiple (two or three) timepoints with the remaining women being deficient at one timepoint only. Almost all women (97.7%) were reportedly taking some form of vitamin D-containing supplement during pregnancy with 6.5% of women taking vitamin D only, 37.4% taking a pregnancy multivitamin only and over half (56.1%) taking a vitamin D supplement plus a pregnancy multivitamin. Of the five women who were not taking any form of vitamin D-containing supplement, one woman was VDD and this was at early pregnancy only. Over one third (35.3%) of women were born in Asia / South Asia and Middle East/ Africa / South America and therefore classified as ‘high risk’ for VDD. Of these ‘high risk’ women, 70.5% were reportedly taking a vitamin D supplement plus a pregnancy multivitamin and 21.8% were taking just a pregnancy multivitamin.

**Table 2:** Mean maternal vitamin D levels across pregnancy and use of vitamin D supplements

Vitamin D measure	mean ± SD or n (%)
Serum 25(OH) D (nmol/L)	
Early pregnancy (*n* = 185)	83.8 ± 22.6
Mid pregnancy (*n* = 208)	96.5 ± 28.9*
Late pregnancy (*n* = 162)	100.8 ± 30.8**
Vitamin D deficiencyª	
Early pregnancy (*n* = 185)	12 (6.5%)
Mid pregnancy (*n* = 208)	13 (6.3%)
Late pregnancy (*n* = 162)	11 (6.8%)
Taking pregnancy vitamin D supplement (*n* = 219)	214 (97.7%)
vitamin D supplement only (*n* = 214)	14 (6.5%)
Pregnancy multivitamin only (*n* = 214)	80 (37.4%)
vitamin D supplement plus pregnancy multivitamin (*n* = 214)	120 (56.1%)
**Vitamin D supplement use by region of birth (*n* = 219)**	**mean ± SD or n (%)**
High risk for vitamin D deficiency^b^	78 (35.3%)
vitamin D supplement only	5 (6.4%)
Pregnancy multivitamin only	17 (21.8%)
vitamin D supplement plus pregnancy multivitamin	55 (70.5%)
Not taking any form of VD supplement	1 (1.3%)
Not high risk for vitamin D deficiency^c^	145 (64.7%)
vitamin D supplement	9 (6.3%)
Pregnancy multivitamin only	63 (44%)
vitamin D supplement plus pregnancy multivitamin	65 (45.5%)
Not taking any form of VD supplement	4 (2.6%)

*Paired-samples t-test result *p* < 0.05 comparing mean (SD) of early pregnancy vitamin D and mid pregnancy vitamin D **Paired-samples t-test result *p* < 0.05 comparing mean (SD) of early pregnancy vitamin D and late pregnancy vitamin D

ªVitamin D deficiency defined as serum 25(OH)D < 50 nmol/L

^b^Region of birth considered high risk for vitamin D deficiency (includes Asia/ South Asia/ Middle East/ Africa/ South America);

^c^Region of birth considered not high risk for vitamin D deficiency (includes Australia/New Zealand/ Europe/ UK/ Canada)

### Associations of maternal vitamin D with birthweight, macrosomia and large for gestational age

Associations of maternal vitamin D across pregnancy with neonatal birthweight are presented in Table 3. In the unadjusted analyses, there was a statistically significant association between early pregnancy plasma 25(OH)D and birth length (0.02 cm, 95% CI, 0.00, 0.03, *p* = 0.020), however the association was no longer statistically significant when the model was adjusted for important confounders (0.01 cm, 95% CI, 0.00, 0.02, *p* = 0.081). In the adjusted models, no statistically significant associations were found between plasma 25(OH)D across pregnancy and birth length, birthweight, LGA and macrosomia. Further, no statistically significant associations were found between VDD (plasma 25(OH)D < 50 nmol/L) and any neonatal outcomes at early, mid or late pregnancy. A separate sub-group analysis was conducted for women diagnosed with GDM (9.6%) which assessed correlations of plasma vitamin D with birthweight. In the unadjusted model there was a positive relationship between early pregnancy vitamin D and birthweight (r = 0.56, *n* = 20, *p* = 0.011) but no significant correlation with mid or late pregnancy vitamin D and birthweight (data not shown). We were unable to conduct any adjusted regression analysis for women with GDM due to the small proportion of available cases.

**Table 3:** Associations of maternal vitamin D across pregnancy with neonatal outcomes

**Vitamin D measure**ª	**Neonatal outcome**	**β (95% CI)**	**OR (95% CI)**	***P*-value**
**Early pregnancy**				
Crude	Birth length (cm)	0.02 (0.00, 0.03)		**0.020**
	Birthweight (g)	2.12 (-1.22, 5.47)		0.212
	LGA		1.01 (0.99, 1.03)	0.433
	Macrosomia		1.01 (0.99, 1.03)	0.415
Adjusted^c^	Birth length (cm)	0.01 (0.00, 0.03)		0.081
	Birthweight (g)	0.37 (-2.52, 3.26)		0.801
	LGA		1.01 (0.99, 1.03)	0.589
	Macrosomia		1.00 (0.98, 1.02)	0.689
**Mid pregnancy**				
Crude	Birth length (cm)	0.01 (0.00, 0.02)		0.054
	Birthweight (g)	0.96 (-1.64, 3.56)		0.468
	LGA		1.00 (0.98, 1.01)	0.512
	Macrosomia		0.99 (0.98, 1.00)	0.195
Adjusted^c^	Birth length (cm)	0.01 (0.00, 0.02)		0.226
	Birthweight (g)	-0.70 (-2.85, 1.45)		0.522
	LGA		1.00 (0.98, 1.01)	0.629
	Macrosomia		0.99 (0.98, 1.01)	0.262
**Late pregnancy**				
Crude	Birth length (cm)	0.01 (-0.01, 0.02)		0.411
	Birthweight (g)	-0.10 (-2.74, 2.55)		0.943
	LGA		0.99 (0.98, 1.01)	0.216
	macrosomia		0.99 (0.98, 1.01)	0.327
Adjusted^c^	birth length (cm)	0.01 (0.00, 0.02)		0.302
	birthweight (g)	-0.04 (-2.17, 2.09)		0.971
	LGA		0.99 (0.98, 1.01)	0.332
	macrosomia		1.00 (0.98, 1.01)	0.555
**Vitamin D deficiency**^b^	**Neonatal outcome**	**β (95% CI)**	**OR (95% CI)**	***P*-value**
**Early pregnancy**				
Crude	birth length (cm)	-0.84 (-2.26, 0.59)		0.248
	birthweight (g)	-159.53 (-470.47, 151.41)		0.313
	LGA		0.63 (0.08, 4.94)	0.656
	macrosomia^d^		-	-
Adjusted^c^	birth length (cm)	0.11 (-1.12, 1.34)		0.858
	birthweight (g)	58.67 (-206.12, 323.46)		0.662
	LGA		0.83 (0.010, 6.92)	0.861
	macrosomia^d^		-	
**Mid pregnancy**				
Crude	birth length (cm)	-1.07 (-2.73, 0.59)		0.204
	birthweight (g)	-144.92 (-481.00, 191.16)		0.395
	LGA		1.27 (0.26, 6.08)	0.769
	macrosomia		1.29 (0.27, 6.10)	0.749
Adjusted^c^	birth length (cm)	-0.05 (-1.36, 1.25)		0.936
	birthweight (g)	128.39 (-134.94, 391.71)		0.337
	LGA		1.22 (0.23, 6.35)	0.813
	macrosomia		1.30 (0.25, 6.81)	0.760
**Late pregnancy**				
Crude	birth length (cm)	-0.06 (-1.66, 1.54)		0.940
	birthweight (g)	110.86 (-221.38, 443.10)		0.510
	LGA		1.61 (0.36, 7.18)	0.530
	macrosomia		1.71 (0.37, 7.93)	0.490
Adjusted^c^	birth length (cm)	-0.15 (-1.45, 1.15)		0.818
	birthweight (g)	91.75 (-181.93, 365.44)		0.508
	LGA		1.43 (0.31, 6.71)	0.647
	macrosomia		1.56 (0.31, 7.81)	0.587

ªSerum 25(OD)D

^b^vitamin D deficiency defined as defined as serum 25(OH)D < 50 nmol/L; birth length and birth weight (continuous outcomes); LGA and macrosomia (binary outcomes); LGA (large for gestational age)

ͨAdjusted models (adjusted for maternal age, gestation length, region of birth and smoking status, for neonatal length and birthweight outcomes, maternal age, region of birth and early pregnancy BMI for macrosomia and LGA)

^d^insufficient cell numbers and precision/ power/ robustness therefore macrosomia omitted from this model

### Associations of maternal vitamin D with gestational weight gain and BMI

Associations of maternal vitamin D across pregnancy with maternal GWG and BMI are presented in Table 4. Both mid and late pregnancy plasma 25(OH)D were negatively associated with late pregnancy BMI (-0.03 kg, 95% CI, -0.06, -0.01, *p* = 0.003 and -0.04, 95% CI, -0.06,—0.01, *p* = 0.02 respectively) in the unadjusted analyses. In the adjusted analyses, no statistically significant associations were found between plasma 25(OH)D at early, mid or late pregnancy with maternal GWG or BMI. Vitamin D deficiency at mid pregnancy was negatively associated with GWG (-3.13 kg, 95% CI, -5.62, -0.65, *p* = 0.014) in the unadjusted analyses and this relationship remained statistically significant in the adjusted model (-2.70 kg, 95% CI, -5.23, -0.17, *p* = 0.037). There were no statistically significant associations found between VDD and at early or late pregnancy with GWG or BMI.

**Table 4:** Associations of maternal vitamin D across pregnancy with maternal gestational weight gain and BMI

**Vitamin D measure**ª	**Weight outcome**	**β (95% CI)**	***P*-value**
**Early pregnancy**			
Crude	GWG (kg)	-0.01 (-0.04, 0.02)	0.497
	late BMI (kg/m^2^)	-0.02 (-0.05, 0.01)	0.201
Adjusted^c^	GWG (kg)	-0.02 (-0.05, 0.02)	0.333
	late BMI (kg/m^2^)	-0.01 (-0.03, 0.00)	0.058
**Mid pregnancy**			
Crude	GWG (kg)	0.01 (-0.01, 0.03)	0.415
	late BMI (kg/m^2^)	-0.03 (-0.06, -0.01)	**0.003**
Adjusted^c^	GWG (kg)	0.00 (-0.02, 0.02)	0.791
	late BMI (kg/m^2^)	-0.01 (-0.01, 0.00)	0.271
**Late pregnancy**			
Crude	GWG (kg)	0.00 (-0.02, 0.03)	0.676
	late BMI (kg/m^2^)	-0.04 (-0.06, -0.01)	**0.002**
Adjusted^c^	GWG (kg)	0.00 (-0.02, 0.02)	0.827
	late BMI (kg/m^2^)	0.00 (-0.02, 0.01)	0.504
**Vitamin D deficiency**^b^	**Weight outcome**	**β (95% CI)**	***P*-value**
**Early pregnancy**			
Crude	GWG (kg)	-1.64 (-4.54, 1.25)	0.263
	late BMI (kg/m^2^)	0.11 (-2.79, 3.02)	0.938
Adjusted^c^	GWG (kg)	-1.32 (-4.22, 1.59)	0.371
	late BMI (kg/m^2^)	-0.48 (-1.72, 0.75)	0.441
**Mid pregnancy**			
Crude	GWG (kg)	-3.13 (-5.62, -0.65)	**0.014**
	late BMI (kg/m^2^)	1.39 (-1.28, 4.06)	0.307
Adjusted^c^	GWG (kg)	-2.70 (-5.23, -0.17)	**0.037**
	late BMI (kg/m^2^)	-0.89 (-2.01, 0.23)	0.117
**Late pregnancy**			
Crude	GWG (kg)	-1.34 (-3.98, 1.31)	0.320
	late BMI (kg/m^2^)	2.45 (-0.39, 5.29)	0.090
Adjusted^c^	GWG (kg)	-0.86 (-3.54, 1.81)	0.525
	late BMI (kg/m^2^)	-0.26 (-1.45, 0.94)	0.671

ªSerum 25(OD)D

^b^vitamin D deficiency defined as defined as serum 25(OH)D < 50 nmol/L; GWG and BMI (continuous outcomes); GWG (gestational weight gain); BMI (body mass index)

^c^Models adjusted for maternal age, region of birth, early pregnancy BMI and education

## Discussion

The main findings from this study were that neither plasma 25(OH)D measures at early, mid or late pregnancy, or VDD (plasma 25(OH)D < 50 nmol/L) were associated with neonatal birth length, birthweight, macrosomia or LGA in a sample of 221 women residing in Melbourne, Australia, attending low-risk antenatal clinics. Furthermore, we found that there was a low prevalence (< 7%) of VDD across pregnancy. This finding likely related to the high proportion of women (98%) who reported taking some form of vitamin D-containing supplements during pregnancy. These findings are important in the context of assessing and understanding in utero environments in shaping early life weight gain, in light of a dramatic increase in global prevalence of childhood obesity throughout the past few decades [65].

Contrary to our hypothesis, we observed no significant associations between plasma 25(OH)D or VDD at any timepoint in pregnancy with birthweight, macrosomia and LGA. Broadly, VDD has been shown to be associated with obesity yet the mechanisms of cause are not well understood [66]. During pregnancy, understanding the relationship is even more complex and it is likely that genetic factors play a role in the interaction between vitamin D and offspring adiposity [31]. This, along with additional modifiable factors such as maternal dietary macronutrient and micronutrient intake, the widespread use of vitamin D supplements amongst nearly all (98%) women in our study and the fact that their mean plasma 25(OH)D concentrations ranged from 84 to 101 nmol/L throughout pregnancy, likely influenced our findings and the relationship between maternal vitamin D and birthweight outcomes more broadly.

Of the limited number of studies that have assessed the relationship of VDD and neonatal growth to date, findings have been mixed [22, 24, 67] and significant associations have been shown to vary by pregnancy trimester. For example, in a study of pregnant women in Spain (*n* = 2358), maternal vitamin D was measured at 13–15 weeks gestation. Deficiency (defined as 25(OH)D < 20 ng/ml or < 69 nmol/L) was evident in 19.6% of women and was shown to predict fetal overweight (fetal weight ≥ 90^th^ percentile) and abdominal adiposity (abdominal circumference ≥ 90^th^ percentile) at birth [31]. In a separate study of Chinese women who had vitamin D assessed during the second (*n* = 11,634) and third trimester of pregnancy (*n* = 6609), median 25(OH)D concentration was found to be ~ 66 nmol/L, an maternal vitamin D in the third but not second trimester was negatively associated with macrosomia but not with birthweight, after adjustment for multiple confounders [67]. Variation in assessment timepoints of vitamin D during pregnancy, difference in the techniques used to analyse 25(OH)D, variation in cut points used to define VDD or variation in adjustment for critical confounders such as season of sample collection likely explain the observed inconsistent results across studies. However, in our study the lack of any significant associations with birthweight, macrosomia and LGA is likely related to the finding that vitamin D status was adequate in the vast majority of women across pregnancy with < 7% observed to have levels below 50 nmol/L, widely regarded as insufficient or deficient.

It is well established that overweight and obesity are independent risk factors for maternal VDD [61, 68] and are components of the screening criteria used to classify women as clinically ‘high risk’ for VDD [66]. While we did not assess pre-pregnancy BMI as part of this study, the assessment of maternal weight gain across pregnancy is an important consideration in the context of monitoring maternal vitamin D status. In our study, we assessed secondary outcomes of maternal weight status and found that plasma 25(OH)D levels at both mid and late pregnancy were negatively associated with late pregnancy BMI in the unadjusted model only, but this did not persist after adjusting for confounders. However, in both the unadjusted and adjusted analyses, mid pregnancy VDD but neither early nor late pregnancy VDD, was negatively associated with total GWG. It is not entirely clear why this relationship was observed at only mid pregnancy and only very few studies to date have assessed the relationship between maternal vitamin D status and total GWG and results have been conflicting [60, 69, 70]. Interestingly in their prospective cohort study of 163 Brazilian women, Figueiredo et al. found that only in women who were overweight at the beginning of pregnancy, but not those who were a healthy weight, vitamin D inadequacy (25(OH)D < 50 nmol/L) in the first and third trimesters, but not in the second trimester, was associated with higher increases in GWG compared to vitamin D adequacy [60]. Assessment of GWG is complex as maternal weight is comprised of multiple components, with fat reserves accounting for roughly one third of the total weight gain. How vitamin D status impacts total GWG, fat mass or other components specifically, or how vitamin D levels impact maternal weight change across pregnancy more broadly is largely uncertain. Yet, possible mechanisms to explain the relationship between VDD and excess weight have included evidence that vitamin D modulates adipogenesis and apoptosis, thereby regulating the growth of adipose tissue [60, 69, 71] and that vitamin D may play a role in decreasing inflammation in adipose tissue [60, 71].

Overall, 9.6% of women in this study were diagnosed with GDM during pregnancy. Further exploring the relationship between VDD with glucose intolerance, insulin resistance and the role of parathyroid hormone as an underlying factor in these associations is important to better understand the impact of vitamin D on development of GDM. As prevalence of GDM is increasing globally [72] and previous research has highlighted a key potential interaction between maternal vitamin D and important glucose parameters during pregnancy [73–75], there is a need for further research in this area.

Maternal vitamin D levels increased significantly across pregnancy from early to mid pregnancy and late pregnancy in our sample of women, independent of season and other relevant confounders. Likewise, of the limited available studies that have assessed vitamin D across three trimesters, in pregnant cohorts with similar rates of supplement usage to the women in our study, highest 25(OH)D levels have been reported in the third trimester [49, 76] even after adjustment for seasonal variation [49]. In a recent longitudinal study of Canadian pregnant women (*n* = 79), vitamin D levels increased significantly from 68 nmol/L to 87 nmol/L and 88 nmol/L across the first, second and third trimesters respectively [49]. In an earlier longitudinal study of Swedish women (*n* = 184), plasma 25(OH)D levels also increased with pregnancy gestation, from 55 nmol/L to 60 nmol/L and 65 nmol/L [76]. As well as use of vitamin D supplementation in pregnancy, physiological adaptations which occur during pregnancy may in part have influenced the observed upward trend of 25(OH)D in our study. For instance, it has been previously reported that the rise in estrogen which occurs naturally with pregnancy progression promotes an increase in vitamin D binding protein as well as active forms of vitamin D, and subsequently a rise in maternal circulating 25(OH)D, irrespective of VD supplement use [49]. This highlights the importance of understanding and considering the physiological adaptations which occur during pregnancy, when assessing vitamin D status and nutritional markers more broadly across pregnancy and interpreting outcomes.

Moreover, mean plasma 25(OH)D levels across early, mid and late pregnancy in our sample were well above the cut point for VDD (< 50 nmol/L), with rates of VDD at each timepoint < 7%. These findings can most likely be attributed to almost all women (98%) reportedly taking a vitamin D-containing supplement during their pregnancy, in the form of a vitamin D specific supplement (7%), a pregnancy multivitamin (37%) or both forms of supplement (56%). The high use of vitamin D-containing supplements in our women may be attributed to the fact that 77% were tertiary educated, and education has been previously associated with high use of multivitamins and vitamin D supplements in pregnancy in high income countries [77]. Elsewhere, recent data has shown that the use of multivitamins and specifically vitamin D -containing supplements across pregnant populations globally varies widely [78–80], although small sample sizes and variations in measurement of adherence make it difficult to compare data across studies. Whether women were actively choosing to take vitamin D or if this was part of clinical management of a pre-existing deficiency was not able to be assessed in this study. Moreover, women may have been unaware they were taking a vitamin D-containing supplement. For example in a survey of 175 pregnant women attending an antenatal clinic in Dublin, 39% of women were taking a pregnancy supplement that knowingly contained vitamin D whilst 56% of women were taking vitamin D as part of their multivitamin supplement but were unaware they were doing so [81].

Whether women in our study taking a vitamin D-containing supplement were recommended to do so by antenatal clinicians after screening women considered ‘high risk’ for deficiency is also an important consideration. Australian recommendations specify that pregnant women considered ‘high risk’ of suboptimal vitamin D (including women born across regions classified as ‘high risk’ e.g. Asia and Africa) be tested, with supplementation advised for deficient women only [82]. In our study, over one third (35%) of women were classified as ‘high risk’ since they were born in Asia / South Asia, the Middle East/ Africa / South America, and indeed almost all (99%) were taking vitamin D-containing supplements. Screening and management of VDD for women from diverse cultural backgrounds is important in the context of ensuring women vulnerable to nutrient deficiencies are adequately supported to achieve best pregnancy outcomes.

Whilst the assessment of offspring weight beyond birth was outside the scope of this study, exploring associations of maternal vitamin D status longitudinally with weight trajectory from birth to early childhood could be an important additional step in further understanding the role of vitamin D in shaping long term offspring weight. Findings from the Southampton Women’s Survey for example, showed that low maternal vitamin D status at 34 weeks gestation (≤ 50 nmol/L) was associated with lower fat mass at birth [83, 84] but with greater fat mass when children were aged 6 years [84]. Despite studies of such design currently lacking, future work in this area would be useful in the overall context of understanding how maternal vitamin D status during pregnancy contributes to future offspring obesity.

There are a number of strengths and limitations of this study. A key strength of this study is that we assessed maternal 25 (OH)D at multiple timepoints across pregnancy and adjusted for important confounders which are known to influence vitamin D levels. We used the gold standard LC/MS/MS method to quantify plasma 25(OH)D [76]. We did not assess physical activity in this study and could not account for this a potential confounder. This is a limitation as physical inactivity is known to be associated with VDD [85]. As this was a retrospective analyses with a relatively small sample size, it was likely underpowered to detect neonatal growth parameters. Specifically, the low number of LGA babies was also a limitation, yet as this study was secondary analyses of the CPO study, the initial study question was not designed to explicitly assess a high number of LGA birth outcomes. This study recruited mostly tertiary educated women (77%) with low-risk pregnancies, with the vast majority having adequate vitamin D levels, and thus the findings cannot be generalised to all cohorts of pregnant women. In particular, we did not recruit non-English speaking women, who may include immigrant populations and those specifically at risk of VDD. Finally, since we did not record dosage of vitamin D supplements taken by the women in our study, we cannot make any conclusions about whether a given dosage of vitamin D was associated with a given change in serum 25(OH)D during pregnancy. Compliance to supplementation is an important consideration for future work, particularly for women who are VDD at the beginning of pregnancy.

## Conclusions

In this cohort of women with low-risk pregnancies in Melbourne, Australia, plasma 25(OH)D levels across pregnancy or maternal VDD were not associated with neonatal birthweight, macrosomia and LGA. Furthermore, the prevalence of VDD deficiency was low (< 7%) in this sample who were predominantly highly educated women with the vast majority (98%) taking some form of vitamin D supplement during pregnancy. Whist there was some observed interaction between vitamin D levels and maternal weight status, further work is needed to better understand the role of vitamin D in maternal GWG. As in utero environments can be highly influential in determining short and long-term offspring obesity, future research among large and diverse pregnant samples is warranted to further understand the role of maternal vitamin D in development of obesity.

## Supplementary Information

**Additional file 1**.

## Data Availability

Data is not publicly available but the datasets used and/or analysed during the current study are available from the corresponding author on reasonable request.

## Abbreviations

VDD: Vitamin D deficiency
LGA: Large for gestational age
GWG: Gestational weight gain
BMI: Body mass index

## References

[1] Zou Z, Yang Z, Yang Z, Wang X, Gao D, Dong Y, Ma J, Ma Y (2019). Association of high birth weight with overweight and obesity in Chinese students aged 6–18 years: a national, cross-sectional study in China. BMJ Open.

[2] Ng M, Fleming T, Robinson M, Thomson B, Graetz N, Margono C, Mullany EC, Biryukov S, Abbafati C, Abera SF (2014). Global, regional, and national prevalence of overweight and obesity in children and adults during 1980–2013: a systematic analysis for the Global Burden of Disease Study 2013. The Lancet.

[3] Ng S-K, Olog A, Spinks AB, Cameron CM, Searle J, McClure RJ (2010). Risk factors and obstetric complications of large for gestational age births with adjustments for community effects: results from a new cohort study. BMC Public Health.

[4] Mohammadbeigi A, Farhadifar F, Soufi Zadeh N, Mohammadsalehi N, Rezaiee M, Aghaei M (2013). Fetal macrosomia: risk factors, maternal, and perinatal outcome. Ann Med Health Sci Res.

[5] Modzelewski J, Pokropek A, Jakubiak-Proć M, Muzyka-Placzyńska K, Filipecka-Tyczka D, Kajdy A, Rabijewski M: Large-for-gestational-age or macrosomia as a classifier for risk of adverse perinatal outcome: a retrospective cross-sectional study. The Journal of Maternal-Fetal & Neonatal Medicine 2021:1–8. 10.1080/14767058.2021.1887127 33602007

[6] Pan X-F, Tang L, Lee AH, Binns C, Yang C-X, Xu Z-P, Zhang J-L, Yang Y, Wang H, Sun X (2019). Association between fetal macrosomia and risk of obesity in children under 3 years in Western China: a cohort study. World Journal of Pediatrics.

[7] Sparano S, Ahrens W, De Henauw S, Marild S, Molnar D, Moreno LA, Suling M, Tornaritis M, Veidebaum T, Siani A (2013). Being macrosomic at birth is an independent predictor of overweight in children: results from the IDEFICS study. Matern Child Health J.

[8] Sacco MR, de Castro NP, Euclydes VLV, Souza JM, Rondó PHC (2013). Birth weight, rapid weight gain in infancy and markers of overweight and obesity in childhood. Eur J Clin Nutr.

[9] Czarnobay SA, Kroll C, Schultz LF, Malinovski J (2019). Mastroeni SSdBS, Mastroeni MF: Predictors of excess birth weight in Brazil: a systematic review. Jornal de Pediatria.

[10] Rao C, Ping F (2021). Second-trimester maternal lipid profiles rather than glucose levels predict the occurrence of neonatal macrosomia regardless of glucose tolerance status: A matched cohort study in Beijing. J Diabetes Complications.

[11] Vargas-Terrones M, Nagpal TS, Barakat R (2019). Impact of exercise during pregnancy on gestational weight gain and birth weight: an overview. Braz J Phys Ther.

[12] Shapiro ALB, Kaar JL, Crume TL, Starling AP, Siega-Riz AM, Ringham BM, Glueck DH, Norris JM, Barbour LA, Friedman JE (2016). Maternal diet quality in pregnancy and neonatal adiposity: the Healthy Start Study. Int J Obes.

[13] Holick MF, Binkley NC, Bischoff-Ferrari HA, Gordon CM, Hanley DA, Heaney RP, Murad MH, Weaver CM (2011). Evaluation, treatment, and prevention of vitamin D deficiency: an Endocrine Society clinical practice guideline. J Clin Endocrinol Metab.

[14] Wei S-Q, Qi H-P, Luo Z-C, Fraser WD (2013). Maternal vitamin D status and adverse pregnancy outcomes: a systematic review and meta-analysis. J Matern Fetal Neonatal Med.

[15] van der Pligt P, Willcox J, Szymlek-Gay EA, Murray E, Worsley A, Daly RM (2018). Associations of maternal vitamin D deficiency with pregnancy and neonatal complications in developing countries: a systematic review. Nutrients.

[16] Tous M, Villalobos M, Iglesias L, Fernández-Barrés S, Arija V (2020). Vitamin D status during pregnancy and offspring outcomes: a systematic review and meta-analysis of observational studies. Eur J Clin Nutr.

[17] Karras SN, Wagner CL, Castracane VD (2018). Understanding vitamin D metabolism in pregnancy: From physiology to pathophysiology and clinical outcomes. Metabolism.

[18] Specker B (2004). Vitamin D requirements during pregnancy. Am J Clin Nutr.

[19] Francis EC, Hinkle SN, Song Y, Rawal S, Donnelly SR, Zhu Y, Chen L, Zhang C (2018). Longitudinal Maternal Vitamin D Status during Pregnancy Is Associated with Neonatal Anthropometric Measures. Nutrients.

[20] Dovnik A, Mujezinović F (2018). The Association of Vitamin D Levels with Common Pregnancy Complications. Nutrients.

[21] Danese E, Pucci M, Montagnana M, Lippi G: Vitamin D deficiency and pregnancy disorders. Journal of Laboratory and Precision Medicine 2019, 5.

[22] Ni M, Zhang Q, Zhao J, Shen Q, Yao D, Wang T, Liu Z (2021). Relationship between maternal vitamin D status in the first trimester of pregnancy and maternal and neonatal outcomes: a retrospective single center study. BMC Pediatr.

[23] Wang H, Xiao Y, Zhang L, Gao Q (2018). Maternal early pregnancy vitamin D status in relation to low birth weight and small-for-gestational-age offspring. J Steroid Biochem Mol Biol.

[24] Ong YL, Quah PL, Tint MT, Aris IM, Chen LW, van Dam RM, Heppe D, Saw S-M, Godfrey KM, Gluckman PD (2016). The association of maternal vitamin D status with infant birth outcomes, postnatal growth and adiposity in the first 2 years of life in a multi-ethnic Asian population: the Growing Up in Singapore Towards healthy Outcomes (GUSTO) cohort study. Br J Nutr.

[25] Aghajafari F, Nagulesapillai T, Ronksley PE, Tough SC, O’Beirne M, Rabi DM (2013). Association between maternal serum 25-hydroxyvitamin D level and pregnancy and neonatal outcomes: systematic review and meta-analysis of observational studies. BMJ : British Medical Journal.

[26] Chen Y, Zhu B, Wu X, Li S, Tao F (2017). Association between maternal vitamin D deficiency and small for gestational age: evidence from a meta-analysis of prospective cohort studies. BMJ Open.

[27] Fang K, He Y, Mu M, Liu K (2021). Maternal vitamin D deficiency during pregnancy and low birth weight: a systematic review and meta-analysis. J Matern Fetal Neonatal Med.

[28] Nimitphong H, Park E, Lee M-J (2020). Vitamin D regulation of adipogenesis and adipose tissue functions. Nutr Res Pract.

[29] McGill A-T, Stewart JM, Lithander FE, Strik CM, Poppitt SD (2008). Relationships of low serum vitamin D3with anthropometry and markers of the metabolic syndrome and diabetes in overweight and obesity. Nutr J.

[30] Tosunbayraktar G, Bas M, Kut A, Buyukkaragoz AH (2015). Low serum 25(OH)D levels are assocıated to hıgher BMI and metabolic syndrome parameters in adult subjects in Turkey. Afr Health Sci.

[31] Morales E, Rodriguez A, Valvi D, Iñiguez C, Esplugues A, Vioque J, Marina LS, Jiménez A, Espada M, Dehli CR (2015). Deficit of vitamin D in pregnancy and growth and overweight in the offspring. Int J Obes.

[32] Esmeraldo CUP, Martins MEP, Maia ER, Leite JLA, Ramos JLS, Gonçalves J, Neta CM, Suano-Souza FI, Sarni ROS (2019). Vitamin D in Term Newborns: Relation with Maternal Concentrations and Birth Weight. Ann Nutr Metab.

[33] Yilmaz S, Aktulay A, Demirtas C, Engin-Ustun Y (2015). Low cord blood serum levels of vitamin D: cause or effect of fetal macrosomia?. Clin Exp Obstet Gynecol.

[34] Wen J, Kang C, Wang J, Cui X, Hong Q, Wang X, Zhu L, Xu P, Fu Z, You L (2018). Association of maternal serum 25-hydroxyvitamin D concentrations in second and third trimester with risk of macrosomia. Sci Rep.

[35] Palacios C, De-Regil LM, Lombardo LK, Peña-Rosas JP (2016). Vitamin D supplementation during pregnancy: Updated meta-analysis on maternal outcomes. J Steroid Biochem Mol Biol.

[36] Eggemoen ÅR, Falk RS, Knutsen KV, Lagerløv P, Sletner L, Birkeland KI, Jenum AK (2016). Vitamin D deficiency and supplementation in pregnancy in a multiethnic population-based cohort. BMC Pregnancy Childbirth.

[37] Lips P, van Schoor NM, de Jongh RT (2014). Diet, sun, and lifestyle as determinants of vitamin D status. Ann N Y Acad Sci.

[38] Larqué E, Morales E, Leis R, Blanco-Carnero JE (2018). Maternal and Foetal Health Implications of Vitamin D Status during Pregnancy. Ann Nutr Metab.

[39] De Guingand DL, Ellery SJ, Davies-Tuck ML, Dickinson H (2019). Creatine and pregnancy outcomes, a prospective cohort study in low-risk pregnant women: study protocol. BMJ Open.

[40] Monash Health [https://monashhealth.org/services/maternity/]

[41] Obesity: Definitions and Measurement [https://ourworldindata.org/obesity#definitions-measurement]

[42] Nordman H, Jääskeläinen J, Voutilainen R (2020). Birth Size as a Determinant of Cardiometabolic Risk Factors in Children. Hormone Research in Paediatrics.

[43] Gaudet L, Ferraro ZM, Wen SW, Walker M (2014). Maternal Obesity and Occurrence of Fetal Macrosomia: A Systematic Review and Meta-Analysis. Biomed Res Int.

[44] Biratu AK, Wakgari N, Jikamo B (2018). Magnitude of fetal macrosomia and its associated factors at public health institutions of Hawassa city, southern Ethiopia. BMC Res Notes.

[45] Martín-Calvo N, Goni L, Tur JA, Martínez JA (2022). Low birth weight and small for gestational age are associated with complications of childhood and adolescence obesity: Systematic review and meta-analysis. Obes Rev.

[46] de Onis M, Habicht JP (1996). Anthropometric reference data for international use: recommendations from a World Health Organization Expert Committee. Am J Clin Nutr.

[47] Fenton TR, Sauve RS (2007). Using the LMS method to calculate z-scores for the Fenton preterm infant growth chart. Eur J Clin Nutr.

[48] Fenton TR, Kim JH (2013). A systematic review and meta-analysis to revise the Fenton growth chart for preterm infants. BMC Pediatr.

[49] Savard C, Bielecki A, Plante A-S, Lemieux S, Gagnon C, Weiler HA, Morisset A-S: Longitudinal Assessment of Vitamin D Status across Trimesters of Pregnancy. The Journal of Nutrition 2021. 10.1093/jn/nxab060 PMC8245879 33830266

[50] Lips P (2007). Relative Value of 25(OH)D and 1,25(OH)2D Measurements. J Bone Miner Res.

[51] Martinaityte I, Kamycheva E, Didriksen A, Jakobsen J, Jorde R (2017). Vitamin D Stored in Fat Tissue During a 5-Year Intervention Affects Serum 25-Hydroxyvitamin D Levels the Following Year. J Clin Endocrinol Metab.

[52] Oliveri B, Mastaglia SR, Brito GM, Seijo M, Keller GA, Somoza J, Diez RA, Di Girolamo G (2015). Vitamin D3 seems more appropriate than D2 to sustain adequate levels of 25OHD: a pharmacokinetic approach. Eur J Clin Nutr.

[53] Atef SH (2018). Vitamin D assays in clinical laboratory: Past, present and future challenges. J Steroid Biochem Mol Biol.

[54] Vitamin D Standardization Program (VDSP) [https://ods.od.nih.gov/Research/vdsp.aspx]

[55] Gill TK, Hill CL, Shanahan EM, Taylor AW, Appleton SL, Grant JF, Shi Z, Grande ED, Price K, Adams RJ (2014). Vitamin D levels in an Australian population. BMC Public Health.

[56] Amrein K, Scherkl M, Hoffmann M, Neuwersch-Sommeregger S, Köstenberger M, Tmava Berisha A, Martucci G, Pilz S, Malle O (2020). Vitamin D deficiency 2.0: an update on the current status worldwide. Eur J Clin Nutr.

[57] Rosen CJ, Abrams SA, Aloia JF, Brannon PM, Clinton SK, Durazo-Arvizu RA, Gallagher JC, Gallo RL, Jones G, Kovacs CS (2012). IOM committee members respond to Endocrine Society vitamin D guideline. J Clin Endocrinol Metab.

[58] White IR, Royston P, Wood AM (2011). Multiple imputation using chained equations: Issues and guidance for practice. Stat Med.

[59] Amberntsson A, Papadopoulou E, Winkvist A, Lissner L, Meltzer HM, Brantsaeter AL, Augustin H (2021). Maternal vitamin D intake and BMI during pregnancy in relation to child’s growth and weight status from birth to 8 years: a large national cohort study. BMJ Open.

[60] Figueiredo ACC, Carrilho TRB, Batalha MA, Farias DR, Barros EG, Kac G (2020). Association between vitamin D status during pregnancy and total gestational weight gain and postpartum weight retention: a prospective cohort. Eur J Clin Nutr.

[61] Bodnar LM, Catov JM, Roberts JM, Simhan HN (2007). Prepregnancy obesity predicts poor vitamin D status in mothers and their neonates. J Nutr.

[62] Wang Y, Ma H, Feng Y, Zhan Y, Wu S, Cai S, Shi Y, Chen Y, Ma L, Jiang Y (2020). Association among pre-pregnancy body mass index, gestational weight gain and neonatal birth weight: a prospective cohort study in China. BMC Pregnancy Childbirth.

[63] Monthly Averages of Daily UV Index In: Ultraviolet Radiation Index Edited by Australian Government ARPaNSA. Australia 2022.

[64] Davidson ZE, Walker KZ, Truby H (2012). Do Glucocorticosteroids Alter Vitamin D Status? A Systematic Review with Meta-Analyses of Observational Studies. J Clin Endocrinol Metab.

[65] Di Cesare M, Sorić M, Bovet P, Miranda JJ, Bhutta Z, Stevens GA, Laxmaiah A, Kengne A-P, Bentham J (2019). The epidemiological burden of obesity in childhood: a worldwide epidemic requiring urgent action. BMC Med.

[66] McAree T (2013). Obesity and Vitamin D Deficiency - Current Concepts on their Impact on Pregnancy. Eur Endocrinol.

[67] Yang W, Jiao M, Xi L, Han N, Luo S, Xu X, Zhou Q, Wang H (2021). The association between maternal fat-soluble vitamin concentrations during pregnancy and infant birth weight in China. Br J Nutr.

[68] McAree T, Jacobs B, Manickavasagar T, Sivalokanathan S, Brennan L, Bassett P, Rainbow S, Blair M (2013). Vitamin D deficiency in pregnancy - still a public health issue. Matern Child Nutr.

[69] Amiri M, Rostami M, Bidhendi-Yarandi R, Fallahzadeh A, Simbar M, Ramezani Tehrani F: Relationship between vitamin D status in the first trimester of the pregnancy and gestational weight gain: a mediation analysis. Archives of Gynecology and Obstetrics 2021. 10.1007/s00404-021-06163-y 34333703

[70] Shakeri M, Jafarirad S (2019). The relationship between maternal vitamin D status during third trimester of pregnancy and maternal and neonatal outcomes: A longitudinal study. International Journal of Reproductive BioMedicine.

[71] Abbas MA (2017). Physiological functions of Vitamin D in adipose tissue. J Steroid Biochem Mol Biol.

[72] Wang H, Li N, Chivese T, Werfalli M, Sun H, Yuen L, Hoegfeldt CA, Elise Powe C, Immanuel J, Karuranga S (2022). IDF Diabetes Atlas: Estimation of Global and Regional Gestational Diabetes Mellitus Prevalence for 2021 by International Association of Diabetes in Pregnancy Study Group’s Criteria. Diabetes Res Clin Pract.

[73] Sirico A, Dell'Aquila M, Tartaglione L, Moresi S, Farì G, Pitocco D, Arena V, Lanzone A: PTH-rP and PTH-R1 Expression in Placentas from Pregnancies Complicated by Gestational Diabetes: New Insights into the Pathophysiology of Hyperglycemia in Pregnancy. Diagnostics (Basel) 2021, 11(8). 10.3390/diagnostics11081356 PMC8394866 34441291

[74] Maghbooli Z, Hossein-Nezhad A, Karimi F, Shafaei AR, Larijani B (2008). Correlation between vitamin D3 deficiency and insulin resistance in pregnancy. Diabetes Metab Res Rev.

[75] Kramer CK, Swaminathan B, Hanley AJ, Connelly PW, Sermer M, Zinman B, Retnakaran R (2014). Vitamin D and parathyroid hormone status in pregnancy: effect on insulin sensitivity, β-cell function, and gestational diabetes mellitus. J Clin Endocrinol Metab.

[76] Lundqvist A, Sandström H, Stenlund H, Johansson I, Hultdin J (2016). Vitamin D Status during Pregnancy: A Longitudinal Study in Swedish Women from Early Pregnancy to Seven Months Postpartum. PLoS ONE.

[77] Bärebring L, Mullally D, Glantz A, Elllis J, Hulthén L, Jagner Å, Bullarbo M, Winkvist A, Augustin H (2018). Sociodemographic factors associated with dietary supplement use in early pregnancy in a Swedish cohort. Br J Nutr.

[78] Asali FF, Tayyem RF, Allehdan SS, Mahfouz IA, Bawadi HA (2020). Use of dietary supplements among pregnant women in the center of Jordan. NFS Journal.

[79] Yamanouchi L, Srinivasan M, Barlow N, Basu A (2021). Level of adherence to vitamin D supplementation guidelines in an antenatal centre in Birmingham, UK, and its effect on biochemical and obstetrical outcomes: a single-centre cross-sectional study. BMJ Open.

[80] Knapik A, Kocot K, Witek A, Jankowski M, Wróblewska-Czech A, Kowalska M, Zejda J, Brożek G (2018). Dietary supplementation usage by pregnant women in Silesia — population based study. Ginekol Pol.

[81] Windrim CM, Crosby DA, Mitchell K, Brophy C, Mahony R, Higgins M: Vitamin D supplementation in pregnancy—a survey of compliance with recommendations. Irish Journal of Medical Science (1971 -) 2018, 187(3):709–712. 10.1007/s11845-017-1707-8 29159790

[82] Australian Government Pregnancy Care Guidelines: vitamin D status [https://www.health.gov.au/resources/pregnancy-care-guidelines/part-g-targeted-maternal-health-tests/vitamin-d-status]

[83] Bi WG, Nuyt AM, Weiler H, Leduc L, Santamaria C, Wei SQ (2018). Association Between Vitamin D Supplementation During Pregnancy and Offspring Growth, Morbidity, and Mortality: A Systematic Review and Meta-analysis. JAMA Pediatr.

[84] Crozier SR, Harvey NC, Inskip HM, Godfrey KM, Cooper C, Robinson SM, Group SWSS (2012). Maternal vitamin D status in pregnancy is associated with adiposity in the offspring: findings from the Southampton Women's Survey. Am J Clin Nutr.

[85] Lee M-J, Hsu H-J, Wu IW, Sun C-Y, Ting M-K, Lee C-C (2019). Vitamin D deficiency in northern Taiwan: a community-based cohort study. BMC Public Health.

